# Assessment of some potentially toxic elements on a watershed-scale through an integrated chemical and biological monitoring framework in the Caatinga-Atlantic Forest ecotone, Northeast Brazil

**DOI:** 10.1007/s11356-026-37568-4

**Published:** 2026-03-04

**Authors:** Kaíque Mesquita Cardoso, Clístenes Williams Araújo do Nascimento, Douglas Gonçalves da Silva, Simone Aparecida da Silva Lins, Maria Eugenia Ortiz Escobar, Pâmalla Graziely Carvalho Morais, Tatiana Reis dos Santos Bastos, Cácio Luiz Boechat

**Affiliations:** 1 Federal Institute of Education, Science and Technology of Northern Minas Gerais, Araçuaí, Minas Gerais 39600-000 Brazil; 2 Graduate Program in Agronomy, State University of Southwest Bahia, Vitória da Conquista, Bahia, 45083-900 Brazil; 3 Federal Rural University of Pernambuco, Recife, Pernambuco 52171-900 Brazil; 4 Federal University of Ceará, Fortaleza, Ceará 60440-554 Brazil; 5 Federal University of Piauí, Campus Profª Cinobelina Elvas, Bom Jesus, Piauí 64900-000 Brazil

**Keywords:** Water resource, Environmental pollution, Bedload sediment, Heavy metals, Macrophytes, *Heteranthera reniformis*, *Eichhornia crassipes*

## Abstract

The concern for water quality is a critical issue in many countries around the world. The monitoring of water resources should be implemented to ensure water management, especially when dealing with already impacted systems. Urbanization is an impactful type of activity that contributes to the release of potentially toxic elements (PTEs) into the ecosystems. The identification of plant species with the ability to tolerate high levels of contaminants could be an alternative to remove contaminants from these environments and to evaluate the plants as bioindicators. The objective of this study was to assess the surface water and sediment quality of the Verruga River and test the aquatic macrophytes’ potential as biomonitors of some PTEs: Cd, Co, Cr, Cu, Mn, Ni, Pb, and Zn. Ten sampling points were included in the study, ranging from the source to the mouth of the river. Samples of surface water, sediments, and macrophytes were collected. In situ and laboratory analyses were performed. Direct readings of PTEs in surface water, extracts from plants and sediments were performed using ICP-OES. Quality reference guides were used. The mobility of the elements can be better visualized by the correlations between the parameters of the sediments than of the water. Concentration of Cd, Cu, Mn, Ni, Pb and Zn in surface water, sediments and aquatic plants reached different levels and some parameters exceeded guidelines on environmental quality. The species *Heteranthera reniformis* and *Eichhornia crassipes* are promising macrophytes for studies on biomonitoring of PTEs in the aquatic environment of the Verruga River.

**Graphic Abstract:**

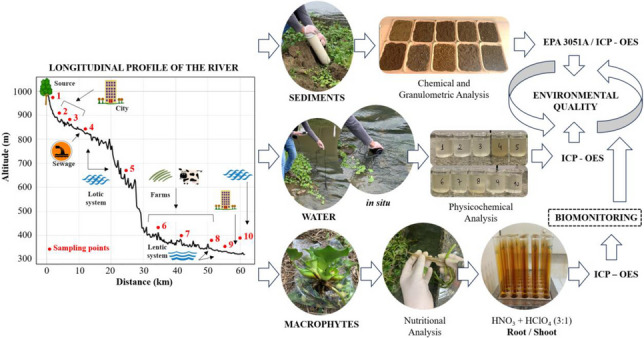

## Introduction

The quality of water resources progressively declines due to excessive use and population growth. Meeting the demands for infrastructure, food production, domestic and industrial supplies, water is a crucial issue for many countries (Sabale et al. 2023; Nunes et al. 2026). Water resource monitoring is imperative, and an approach based on watershed management research is necessary to consider the value of ecosystem services. Thus, an integrated and predictive approach, combined with environmental education and alternatives aiming at improving multiple water uses must be implemented to ensure water resource management in the twenty-first century (Liu et al. 2023a; Tundisi 2008).

The use of macrophytes (aquatic or hydrophytic plants) proves to be a viable option for both biomonitoring and the ability to extract contaminants from water resources, they exhibit great potential for cleaning up the environment, outstanding the economic (low-cost) and ecological (environmentally friendly) benefits of the remediation process (Demarco et al. 2020; Dhir et al. 2009). Macrophytes have been employed to evaluate environmental conditions (Kurniawan et al. 2021). The stream element concentrations are very variable (water metal content should change after a rainfall, or prolonged drought), for this reason, establishing relations (as bioaccumulation and translocation factors) of aquatic plants with the environment may reflect more of an average in the metals over time, (Demarco et al. 2023; Gecheva et al. 2023; Nabi 2021; Sahu et al. 2020).

Aquatic macrophytes serve as sources of food and oxygen for aquatic environments, maintaining prolonged contact with water and sediments. The advantage to using aquatic plants as biomonitors is they establish an equilibrium with the environment in which they are growing. They can exhibit phytoextraction, phytostabilization, phytoaccumulation and phytodegradation properties for some pollutants, processes known as phytoremediation (Ansari et al. 2020; Kurniawan et al. 2021; Sahu et al. 2020; Haghnazar et al. 2021). Phytoremediation is an alternative and innovative method: the first step is the identification of species with the ability to take high levels of pollutants and next step is to understand the main mechanism by which and how the plant removes the contaminant (Demarco et al. 2018, 2020).

The Verruga River watershed, tributary of the important nacional Rio Pardo, has compromised surface water quality and poses ecological risks to the ecosystem (INEMA 2014). According to the SOS Mata Atlântica Foundation (2011), among the water bodies monitored in Brazil, the analysis of the Verruga River in Vitória da Conquista, BA had the worst results. Anthropogenic activities (land occupation and management) can elevate the concentrations of some PTEs (Cu, Cd, Co, Pb and Zn) in the watershed and the soils in specific areas can be classified as moderately enriched and polluted. The soil of the watershed has individual ecological risk concerning Cd (Bastos et al. 2025; Cardoso et al. 2024).

Fifty years ago, the Verruga River watercourse was the main water supply in the region, characterized as a source of recreation and food provision. However, during the twentieth century, public authorities were unable to manage natural elements and flows effectively, and urban expansion proved unsustainable in the long term. The canalization of the urban stretch of the river led to its increasing invisibility. Removed from the sight of the urban population, watercourse pollution reached alarming levels (Vasconcelos and Marta 2022).

The hypothesis of this study is that different species of aquatic macrophytes can be used as biomonitoring agents, revealing pollution patterns and demonstrating the ability to absorb nutrients and potentially toxic elements. Thus, the objective of this study was to assess the environmental quality of the surface water and sediments of the Verruga River and test the absorbing, accumulating and translocating capabilities of the aquatic macrophytes in the river to take some potentially toxic elements (Cd, Co, Cr, Cu, Mn, Ni, Pb, and Zn).

## Materials and methods

### Study area

**Fig. 1:**
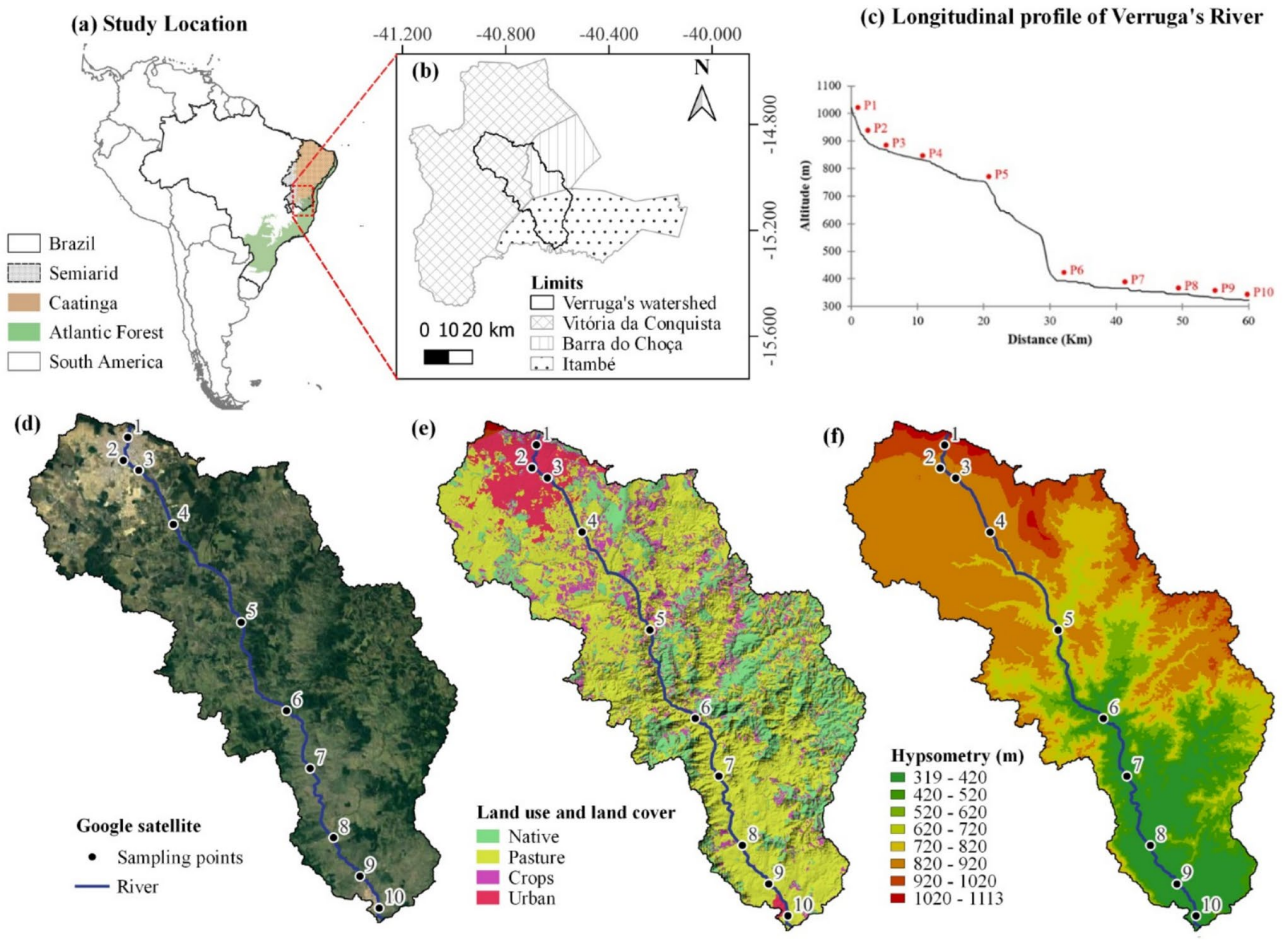
Map of the study area: (**a**) located in the state of Bahia in Brazilian semiarid, South America, (**b**) between the cities of Vitória da Conquista, Barra do Choça and Itambé; (**c**) longitudinal profile of Verruga’s river; (**d**) Google satellite; (**e**) land use and land cover; (**f**) Hypsometry of the Verruga watershed

The Verruga River watershed has a drainage area of 906 km^2^ and spans the cities of Vitória da Conquista, Barra do Choça, and Itambé (Fig. 1). The climate is classified as semi-arid (Zone BSh) with an average annual precipitation of 741 mm, ranging from 367 to 1500 mm, August is the driest month and December is the wettest month. (Alvares et al. 2013; Murta et al. 2005; Rocha 2008).

The Verruga River was once considered an important watercourse for the region, serving human supply, irrigation, and recreation. However, today it is channelized in the second most populous city in the state of Bahia: 396,613 people and receives direct sources of effluents from urban areas as well as diffuse inputs from rural areas (Rocha 2008). The geology of the watershed features an extensive range of detrital cover, originating from weathering actions of mechanical disintegration, bordered by an extensive layer of the Itapetinga complex in the lower portion of the basin (Rocha 2008). Most tributaries in the basin are perennial with a dendritic drainage pattern during heavy rains and a sandy sediment cover, resulting in a natural aptitude for infiltration and groundwater accumulation (Fig. 1d), responsible for the perennialization of the water flow. In the Verruga River watershed, two soil classes predominate: clayey-sandy textured Oxisols in the higher areas of the basin and Ultisols downstream in the lower part (Soil Survey Staff 2014). In its drainage area (Fig. 1e), more than half of the soils are used as pastures (56.24%), followed by native vegetation (24.44%), crops (13.36%), and urban infrastructure (5.92%) (Mapbiomas 2020).

### Sampling points

Ten sampling points (P1 to P10) were selected along the course of the Verruga River based on local characteristics, and their geographical coordinates were obtained using a GPS device (Garmin®). After selection, surface water, sediment, and aquatic macrophyte samples were collected in September 2022 (end of the dry season and beginning of the rainy season): This study was carried out in the dry season because at this time there is less dilution (reduced water flow leads to less dilution of pollutants), consequently, higher concentrations of substances and chemical elements in the water, like metals released into the river become more concentrated in the water column and sediment, which increases their uptake by aquatic plants (Yonis et al. 2024). Generally higher concentrations of metals in water are detected in water bodies during the dry season (Oliveira et al. 2024). The slower growth during the dry season allows for more concentrated accumulation in the plant tissues (Tariq et al. 2025).

Each sampling point had distinct features: P1: Source of the Verruga River located in the Serra do Periperi, in a natural area with an altitude above 1,000 m. The stretch between P1 and P2 is canalized under the city of Vitória da Conquista, the third-largest city in the state of Bahia with approximately 301,000 inhabitants (IBGE 2023) (Fig. 1a); P2: The point where the river ceases to be canalized and has open-air urban effluents on Bartolomeu de Gusmão Avenue; P3: A point located after a small conservation area, however still within the urban perimeter of the city, on Luiz Eduardo Magalhães Avenue; P4: Santa Marta community, which drains part of the effluents from a city sewage treatment plant; P5: Capinal community, with a rural characteristic and a few families; P6: Point in the Serra do Maçal, after a considerable drop in elevation (Fig. 1c and f); P7: Barro Vermelho community, a rural drainage area with diffuse sources; P8: Farm with a westward-flowing tributary and a characteristic lentic system; P9: Before the city of Itambé, also with a characteristic lentic system; and P10: After the city of Itambé at the upstream mouth of the Pardo River. Points P1, P2, P3, P4, P6, P7, and P10 are visibly characterized as lotic systems.

### Surface water, macrophytes and sediment sampling

Surface water samples were collected from established protocol by the Ministry of Health of Brazil: in amber glass bottles (1000 mL) that had been previously rinsed with distilled water and 10% (v/v) HNO_3_ acid solution. The bottles were submerged to a depth of approximately 10 cm from the surface, and the collected material was transported to the laboratory in refrigerated thermal boxes, maintaining a temperature of 5.0 ± 1.0 ºC until the analysis (BRASIL 2013).

**Fig. 2:**
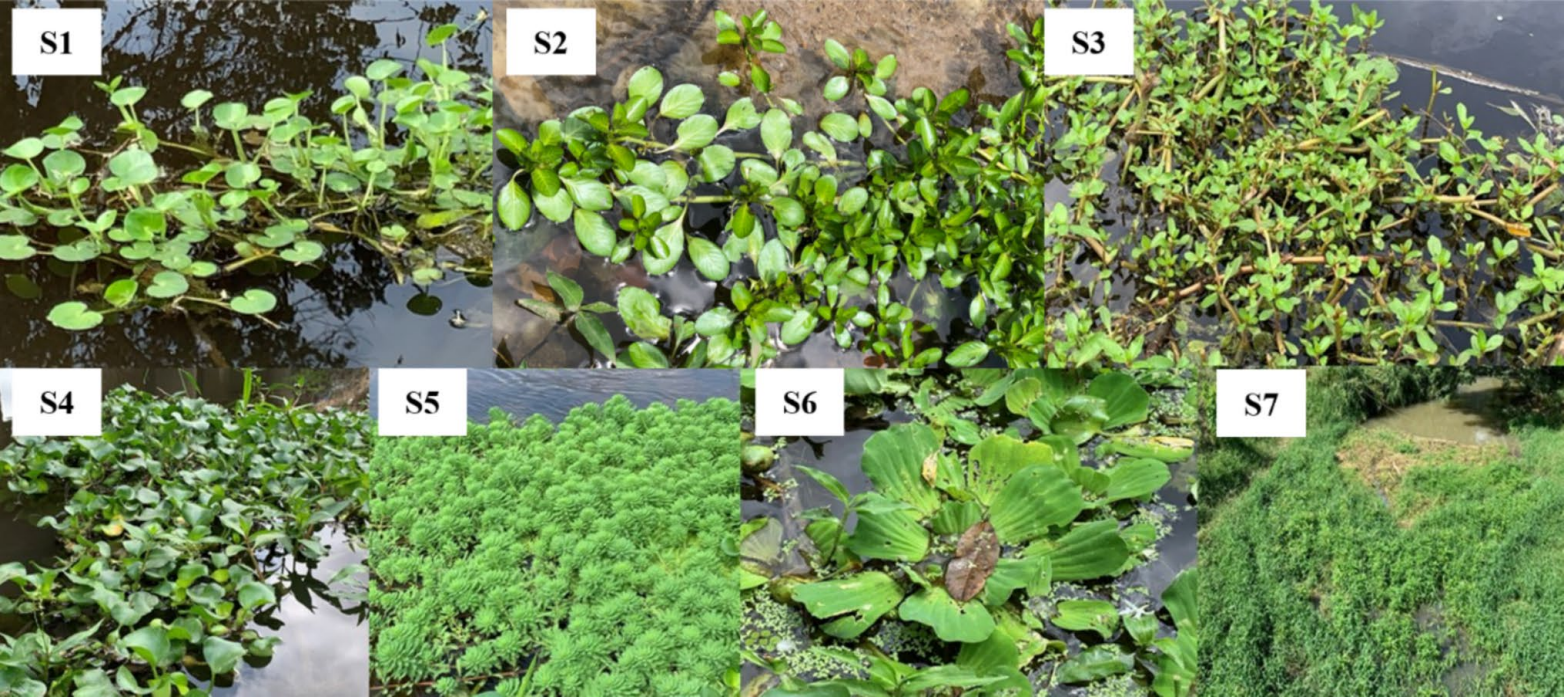
Species identified in the Verruga River, Bahia, Brazil: (**S1**) *H. reniformis*, (**S2**) *L. peploides*, (**S3**) *L. octovalvis*, (**S4**) *P. stratiotes* (**S5**) *M. aquaticum* (**S6**) *E. crassipes* and (**S7**) *P. ferrugineum*

At the sampling points, seven species of aquatic macrophytes were found, distributed across four botanical families (Fig. 2), with respective relative sample frequency values as follows: S1—Pontederiaceae/*Heteranthera reniformis* Ruiz & Pav. (60%), S2—Onagraceae/*Ludwigia peploides* (Kunth) P. H. Raven. (10%), S3—Onagraceae/*Ludwigia octovalvis* (Jacq.) P. H. Raven. (30%), S4—Pontederiaceae/*Eichhornia crassipes* (Mart.) Solms. (30%), S5—Haloragaceae/*Myriophyllum aquaticum* (Vell.) Verdc. (10%), S6—Araceae/*Pistia stratiotes* L. (10%), and S7—Polygonaceae/*Polygonum ferrugineum* Wedd. (10%). The scientific names of the macrophyte samples were verified from the Tropicos Collection (2023).

The plants were manually collected in triplicate within sub-locations (10 m transect) for each species at each collection point, separating roots and shoots. Subsequently, they were stored in sterile polyethylene bags and immediately sent to the laboratory. All samples were washed with tap water, followed by rinsing with distilled water. The roots were immersed in a 1% (v/v) HNO_3_ acid solution for the removal of elements adhered to the root surface, then washed with distilled water (Araújo et al. 2007; Döll et al. 2024; Ivanov et al. 2024; Shi et al. 2024).

Three single bedload sediment samples were collected using PVC pipes in sub-locations (10 m transect) at a depth of 0.0–0.10 m to form a composite sample at each point. In the laboratory, the samples were air-dried, crumbled, and sieved through screens with a 10-mesh opening (< 2.0 mm).

### On-site, laboratory analyses and quality control

At each point, in situ readings of the parameters dissolved oxygen (DO) and water temperature (T) were taken using a self-calibrating Politerm meter (POL-60). In the laboratory, water samples were analyzed for electrical conductivity (EC), total dissolved solids (TDS) (CAAL MCA150), pH (MS Tecnopon mPA210), turbidity (turb) (Tecnopon TB1000), and concentrations of Ca, Mg, K, and Na according to Brazil (2006). Subsequently, the samples were filtered using a vacuum system with the aid of a Büchner funnel, a kitassato, and a quantitative filter paper (unifil C42 blue stripe) and acidified with HNO_3_ (Merck PA) to pH < 2 for the direct determination of metals by inductively coupled plasma optical emission spectrometry (ICP-OES, Perkin Elmer 7000 DV). Blank assays with ultrapure deionized water (Milli-Q, 18.2 MΩ. cm) were used in the analysis batches (ANA 2011; Baird et al. 2017).

Following the methodology described in Embrapa (2009), samples of shoot and root parts of macrophytes were air-dried in a forced-air circulation oven at 60 ºC until a constant weight was obtained. They were then ground in a Wiley-type mill made of stainless steel and subjected to wet digestion with HNO_3_ + HClO_4_ acid solution (3:1 v/v), both of high analytical purity (Merck PA): the combination of these acids provides a strong oxidizing power, which is essential for the complete decomposition of plant tissue samples, which in a closed system can cause technical problems, as like possibility of explosion due to vapor buildup (0.50 ± 0.001 g of sample in a digestion tube, 8 mL of the acid solution was added. The mixture was left to stand cold for a period of 3 h and then heated in a digestion block inside the fume hood, gradually increasing the temperature to 120 °C, maintaining it until the complete release of brown NO_2_ vapors. Once finished, the temperature was increased to 200 °C and maintained until the complete release of white HClO_4_ vapors) (Uddin et al. 2016). The extracts were filtered (CT REF42 blue stripe) and transferred to certified 25 mL volumetric flasks (NBR ISO/IEC), and made up to volume with ultrapure water (Milli-Q, 18.2 MΩ. cm). Ten blanks assays were repeated along with the extracts, and the concentrations of Fe, Mn, Zn, Cu, Ni, Co, Cr, Pb, and Cd were determined by ICP-OES/Optima 7000, Perkin Elmer. with an injection system through an automatic sampler. The operational parameters followed the manufacturer's recommendation. For the evaluation of the nutritional status of the plants, the concentrations of Ca, Mg, P, K, S, Cu, Mn, Fe, and Zn were determined according to the methodology described in Embrapa (2009; 2017). ICP-OES analysis results for the extracts were compared with certificate reference plants by the National Institute of Standards and Technology—NIST. Standard Reference Material – SRM 1570a (Spinach leaves) was used and the recovery rates of Cd, Co, Cr, Cu, Mn, Ni, Pb and Zn range 82–96%. Also, blank samples (*n* = 10) were used for quality control of analyses.

The chemical analyses and sediment particle size determination followed the methodology described in Embrapa (2017). The pH was determined in H_2_O (1:2.5 v/v sediment/solution), the available contents of P, K, Ca, and Mg were chemically characterized by the Mehlich-1 extractor (HCl and H_2_SO_4_), Ca, Mg, and Al by titration after extraction with KCl (1 mol L^−1^). The potential acidity (H + Al) was extracted by calcium acetate at pH 7.0, and total organic carbon was determined by the Walkley–Black method. Additionally, cation exchange capacity (CEC) and sum of bases (SB) were determined to establish the base saturation of the samples (V%), and particle size was determined using the pipette method. The concentrations of the elements Cd, Co, Cu, Cr, Fe, Mn, Ni, Pb, and Zn were determined by.

The extraction of Cd, Co, Cr, Cu, Fe, Mn, Ni, Pb and Zn was performed using the 3051 A EPA method (United States Environmental Protection Agency – USEPA 2007) as suggested by the National Council for the Environment (Conselho Nacional do Meio Ambiente—CONAMA 420/2009 – Brasil 2009). The microwave oven heating program in a closed system and the quality of the methodology (certificate reference materials—SRM 2709a, San Joaquin soil) can be observed in previous studies (Cardoso et al. 2023a, 2024). The tubes were maintained in a closed system (Milestone/Ethos Easy advanced microwave oven) for 20'' to reach 175 °C and 780 W. This temperature was maintained for a further 4′ 30''.

The detection limit (LOD): Eq. 1 and quantification limits (LOQ): Equation. were determined by the method based on analytical curve parameters (Cardoso et al. 2024; Ribani et al. 2004). Where “s” represents the standard deviation of the response from ten blank readings, and “S” represents the slope of the line equation.

1 $$LOD=3.3 \frac{s }{S}$$

2 $$LOQ=10 \frac{s }{S}$$

Determined recovery (%): Cd (95); Co (62); Cr (43); Cu (78); Fe (78); Mn (66); Ni (57); Pb (46) and Zn (61). Recovery base leaching (%): Cd (86); Co (76); Cr (105); Cu (96); Fe (112); Mn (83); Ni (75); Pb (87) and Zn (79). Detection limits (LOD) mg L^−1^: 0.0006 (Fe); 0.00009 (Mn); 0.004 (Pb); 0.0002 (Cd); 0.0006 (Zn); 0.006 (Co); 0.00075 (Cr); 0.003 (Cu); 0.001 (Ni) and 0.004 (As). Quantification limits (LOQ), based on the analytical curve (mg L^−1^): 1.00 (Al), 0.005 (As); 0.002 (Cd); 0.005 (Co); 0.01 (Cr); 0.01 (Cu); 0.50 (Fe); 0.10 (Mn); 0.02 (Ni, Pb and Zn) (Cardoso et al. 2024).

### Biomonitoring by phytoaccumulation

The bioconcentration factor (BCF) > 1 demonstrates the bioaccumulation capacity of macrophytes, while BCF < 1 indicates a limitation in the accumulation capacity. The bioaccumulation factor (BCF) of metals in aquatic macrophytes was calculated using Eq. 3 (Demarco et al. 2023; Fawzy et al. 2012; Krupnova et al. 2018; Sahu et al. 2020):

3 $$BCF= \frac{C\, root \,macrophyte }{C\, sediment}$$

where, C = concentration of the element in the roots (mg kg^−1^) of the macrophytes and in the sediment (mg kg^−1^).

The translocation factor (TF) > 1, and BCF > 1 show the phytoextraction ability, while TF < 1, and BCF > 1 can be considered as phyto-stabilizing (phyto-immobilizing) capacity (Haghnazar et al. 2021). The TF was calculated using Eq. 4 (Demarco et al. 2023; Mazej and Germ 2009):

4 $$TF= \frac{C\, root \,macrophyte }{C\, shoot\, macrophytes}$$

where, C = Concentration of the element in the plant (roots/shoot parts) (mg kg^−1^).

### Statistical analysis

Univariate (mean, median and standard deviation) and bivariate (correlation analyses) statistical analyses were conducted. The data underwent descriptive statistics. Graphical representations in the form of scatters and lines were used to verify the spatial variation of PTE between collection points, both for surface water and sediments. Violin plots and box plots were employed, along with Spearman correlation (the data did not follow a normal distribution, a non-parametric statistic was used). GraphPad Prism 5 and IBM SPSS software were used.

## Results

### Environmental quality and attributes distribution

The surface water samples collected along the course of the Verruga River showed variation in the quality parameters (Table 1), with average results and standard deviation expressed (*n* = 10): pH = 7.0 ± 1.0, EC = 164.3 ± 22.3 µS cm^−1^, turbidity = 15.9 ± 10.7 NTU, TDS = 147.2 ± 80.4 mg L^−1^, DO = 8.0 ± 3.5 mg L^−1^, and T = 21.4 ± 1.3 ºC. The lowest pH value (4.5) was observed at sampling point P3, and the highest (8.0) at P5. Point P4 recorded the highest values for turbidity (45.6 µS cm^−1^) and TDS (284.2 mg L^−1^), and P6 had the highest DO value (13.1 mg L^−1^). Points P9 and P10 had the highest EC values (174.1 and 175.6 µS cm^−1^, respectively) and the lowest DO values (3.4 and 1.8 mg L^−1^). The point located at the source (P1) showed the lowest values for EC (101.8 µS cm^−1^) and TDS (57.3 mg L^−1^).

**Table 1:** Parameters analyzed in surface water and sediment at the 10 collection points of the Verruga River

Measured parameters		P1	P2	P3	P4	P5	P6	P7	P8	P9	P10	Mean	Median	SD
		Water												
pH_H2O_	1:2.5	7.4	6.6	4.5	7.9	8.0	7.8	7.4	7.1	6.9	6.7	7.0	7.2	1.0
EC	us cm^−1^	101.8	172.5	169.2	164.3	166.2	169.5	175.0	175.0	174.1	175.6	164.3	171.0	22.3
Turb	NTU	15.1	7.5	9.9	45.6	13.7	12.4	12.3	15.6	13.8	13.3	15.9	13.5	10.7
Ca	meq L^−1^	0.30	0.70	0.90	0.80	0.80	0.70	0.50	0.30	0.60	0.60	0.60	0.70	0.20
Mg	meq L^−1^	0.20	0.60	0.90	0.70	0.60	0.70	0.40	0.30	0.70	0.50	0.60	0.60	0.20
K	meq L^−1^	0.07	0.25	0.25	0.55	0.47	0.41	0.22	0.10	0.22	0.17	0.27	0.24	0.16
Na	meq L^−1^	0.85	2.6	3.6	3.1	3.4	3.0	1.8	0.80	1.9	1.1	2.2	2.3	1.1
TDS	mg L^−1^	57.3	126.2	165.7	284.2	260.6	215.1	102.2	95.6	82.2	82.6	147.2	114.2	80.4
DO	mg L^−1^	9.1	6.4	8.9	7.4	9.1	13.1	12.0	9.1	3.4	1.8	8.0	9.0	3.5
T	°C	18.6	22.7	21.6	22.6	20.7	20.8	20.5	21.7	22.4	22.0	21.4	21.7	1.3
		Sediment												
pH_H2O_	1:2.5	7.0	6.9	6.5	6.1	6.6	6.9	6.7	7.3	6.8	7.6	6.9	6.9	0.39
H + Al	cmol_c_ dm^−3^	0.67	0.67	0.64	0.67	0.84	0.67	0.65	0.65	0.69	0.89	0.70	0.67	0.09
Ca	cmol_c_ dm^−3^	4.2	3.9	7.1	1.9	1.2	3.2	4.3	4.5	6.5	5.5	4.2	4.3	1.9
Mg	cmol_c_ dm^−3^	0.30	0.50	7.4	1.4	1.0	1.9	3.1	2.4	3.6	2.1	2.4	2.0	2.1
K	cmol_c_ dm^−3^	0.08	0.10	0.10	0.17	0.07	0.97	0.36	0.09	0.18	0.43	0.26	0.14	0.28
SB	cmol_c_ dm^−3^	4.6	4.5	14.6	3.5	2.3	6.1	7.8	7.0	10.3	8.0	6.9	6.5	3.6
CEC	cmol_c_ dm^−3^	5.3	5.2	15.2	4.1	3.1	6.7	8.4	7.7	10.9	8.9	7.6	7.2	3.6
P	mg dm^−3^	9.1	40.0	6.7	110.4	98.8	115.8	108.0	90.9	90.6	109.9	78.0	94.9	42.7
V%	%	87.2	87.1	95.8	83.8	73.0	90.1	92.3	91.5	93.7	90.0	88.5	90.1	6.5
OM	g kg^−1^	15.5	9.0	1.0	5.8	3.0	15.0	13.7	10.2	16.7	9.0	9.9	9.7	5.4
Clay	g kg^−1^	131.0	152.0	302.0	58.0	51.0	90.0	115.0	101.0	201.0	114.0	131.5	114.5	74.1
Silte	g kg^−1^	24.0	41.0	206.0	43.0	38.0	28.0	67.0	78.0	199.0	54.0	77.8	48.5	67.8
Sand	g kg^−1^	846.0	807.0	492.0	899.0	910.0	882.0	818.0	821.0	600.0	833.0	790.0	827.0	136.1

*EC* electric conductivity; *Turb*. Turbidity; *TDS*, total dissolved solids; *DO* dissolved oxygen; *T* temperature; *H* + *Al* acidity potential; *SB* sum of bases; *CEC* cation exchange capacity; *V* base saturation; *OM* organic matter

Regarding the macronutrients present in the surface water samples (Table 1), the average results and standard deviation are expressed in meq L^−1^: Ca (0.62 ± 0.20), Mg (0.56 ± 0.20), K (0.27 ± 0.20), and Na (2.2 ± 1.1). At P1, the lowest concentrations were observed for Ca (0.30), Mg (0.20), and K (0.07), and at P8 for Ca (0.30) and Na (0.80). Meanwhile, at P3, the highest concentrations of Ca (0.90), Mg (0.90), and Na (3.6) were observed, and at P4 for K (0.55).

**Fig. 3:**
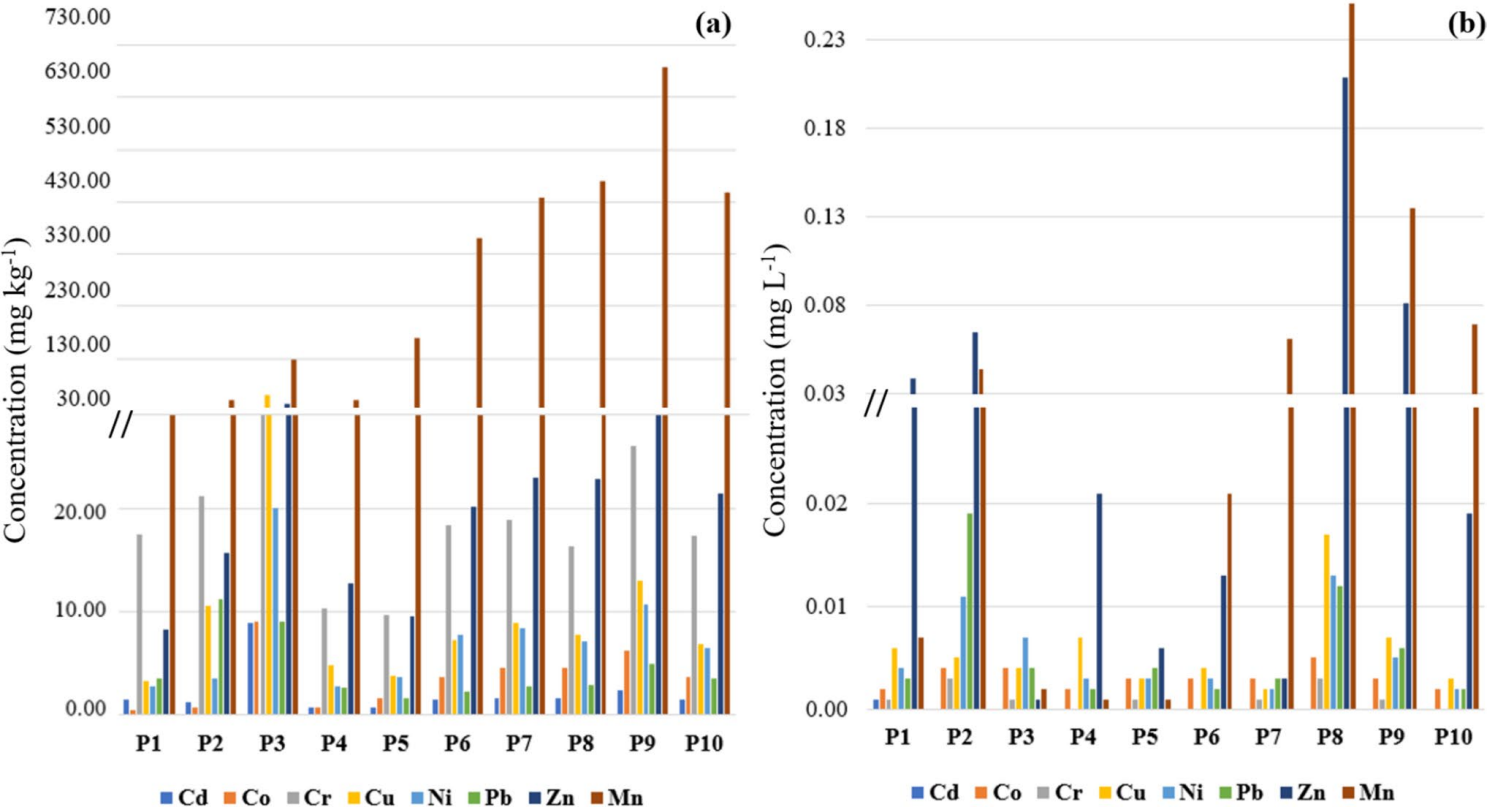
Spatial distribution of potentially toxic elements in samples collected from the Verruga River, sediments (**a**) and surface water (**b**)

Figure 3b shows the spatial distribution of PTEs values in the surface water, in descending order (mg L^−1^): Mn (0.06 ± 0.08) > Zn (0.05 ± 0.06) > Cu (0.01 ± 0.00) > Pb (0.01 ± 0.01) > Ni (0.005 ± 0.004) > Co (0.003 ± 0.001) > Cr (0.001 ± 0.001) > Cd. The highest concentrations for the PTEs were observed at P8, Mn (0.26), Zn (0.21), Cu (0.02), Ni (0.01), Co (0.005), Cr (0.003), except for Pb (0.02) and Cd (0.001 mg L^−1^), which were observed at P2 and P1, respectively. P1 was the only location where the presence of Cd was detected in the surface water samples. The element Fe had 0.40 ± 0.38 mg L^−1^ with P8 the highest value (1.3 mg L^−1^).

### Sediments

The PTEs quantified in sediments, in descending order (mg kg^−1^): Mn (283.0 ± 227.1) > Zn (21.0 ± 10.7) > Cr (18.9 ± 6.9) > Cu (12.6 ± 16.6) > Ni (7.4 ± 5.2) > Co (3.5 ± 2.8) > Pb (4.5 ± 3.7) > Cd (2.2 ± 2.4) (Fig. 3a). The highest concentrations in spatial distribution varied among points: P3 for Cd (8.9), Co (9.1), Cr (32.9), Cu (59.1), Fe (50,000.0), Ni (20.2), and Zn (43.8), P2 for Pb (11.3) and P9 for Mn (687.0), and Fe (16,167.5 ± 12,629.4).

The sediment quality parameters are presented in Table 1 and expressed as means and standard deviations: pH = 6.9 ± 0.39; OM = 9.9 ± 5.4 g kg^−1^; SB = 6.9 ± 3.6 cmol_c_ dm^−3^; H + Al = 0.7 ± 0.09 cmol_c_ dm^−3^; CEC = 7.6 ± 3.6 cmol_c_ dm-3; V% = 88.5 ± 6.5%; clay = 131.5 ± 74.1 g kg^−1^; silt = 77.8 ± 67.8 g kg^−1^; and sand = 790.8 ± 136.1 g kg^−1^. The highest values were observed at P10 (pH = 7.6 and H + Al = 0.89 cmol_c_ dm^−3^), P9 (OM = 16.7 g kg^−1^), and P3 (SB = 14.6 cmol_c_ dm^−3^, CEC = 15.2 cmol_c_ dm^−3^, clay = 302 g kg^−1^, and V = 95.80%). Meanwhile, the lowest values were observed at P3 (H + Al = 0.64 cmol_c_ dm^−3^), P4 (pH = 6.1 and OM = 5.8 g kg^−1^), and P5 (SB = 2.3 cmol_c_ dm^−3^, CEC = 3.1 cmol_c_ dm-3, clay = 51 g kg^−1^, and V = 73.0%).

For macronutrient concentrations (Table 1), the means followed by standard deviations are expressed in cmol_c_ dm^−3^: Ca (4.2 ± 1.9); Mg (2.4 ± 2.1); and K (0.26 ± 0.28), and P in mg dm^−3^ (78.0 ± 42.7). The lowest concentrations were represented by point P5: Ca (1.2 cmol_c_ dm^−3^) and K (0.07 cmol_c_ dm^−3^); point P1 for Mg (0.30 cmol_c_ dm^−3^); and points P3 for P (6.7 mg dm^−3^). The highest concentrations were observed at P3 with Ca (7.1 cmol_c_ dm^−3^); Mg (7.4 cmol_c_ dm^−3^); and P6 with K (0.97 cmol_c_ dm^−3^) and P (115.8 mg dm^−3^).

### Aquatic macrophytes

**Fig. 4:**
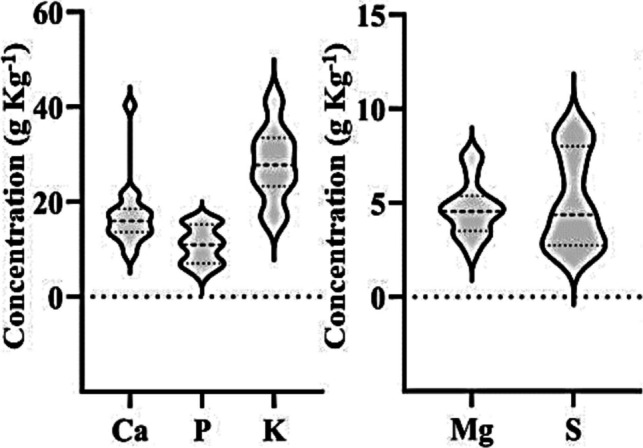
ViolinPlot of macronutrient concentrations in aquatic macrophytes collected from the Verruga River, Bahia, Brazil

The concentrations of macronutrients (g kg^−1^) found in the leaves of aquatic macrophytes, in descending order with means followed by standard deviations: K (27.9 ± 7.7) > Ca (17.2 ± 7.1) > P (10.8 ± 3.9) > S (5.1 ± 2.5) > Mg (4.7 ± 1.5) (Fig. 4). The highest concentrations (g kg^−1^) were observed in the species *H. reniformis* (S1) at different collection sites: P2 (S = 9.2); P4 and P5 (P = 15.9); and P6 (K = 41.9), of Ca (40.3) and Mg (7.5) at P9 in the species *P. stratiotes* (S6).

The concentrations of PTEs in plant tissues varied for both species (S) and collection points (P) according to Table 2. The mean concentrations ± standard deviation in roots, in descending order, were (mg kg^−1^): Mn (1,622.6 ± 1,547.2) > Zn (123.2 ± 76.7) > Cu (20.4 ± 10.5) > Ni (19.9 ± 9.0) > Co (12.7 ± 11.3) > Cr (5.4 ± 3.5) > Pb (4.3 ± 3.5) > Cd (0.56 ± 0.53), and the element Fe 10,348.9 ± 7,050.0 mg kg^−1^. In the species *H. reniformis* (S1), the highest concentrations for PTEs were found at sampling points: P2 (Pb = 14.7 and Fe = 30,006.1), P3 (Cd = 1.9; Cr = 17.7 and Cu = 46.2), P4 (Co = 41.5), P5 (Ni = 40.3), and P6 (Mn = 5,621.4). In the species *L. octovalvis* (S3) collected at point P7, the highest concentration of Zn in roots (379.2) was observed, and the lowest concentrations of Co and Mn (0.4 and 76.5 mg kg^−1^, respectively) were in S1 at P1, for Cr and Ni (4.9 and 2.2 mg kg^−1^, respectively) in S4 at P6, for Zn (41.9 mg kg^−1^) in S7 at P8, and for Cd, Pb, Cu, and Fe (0.05, 0.31, 8.8, and 3,322.3, respectively) in S5 at P10 (Table 2).

**Table 2:** Potentially toxic elements in tissues of aquatic macrophyte species at collection points in the Verruga River: Point (P) and Specie (S)

P	S	Roots									Shoot								
		Cd	Co	Cr	Ni	Pb	Cu	Zn	Mn	Fe	Cd	Co	Cr	Ni	Pb	Cu	Zn	Mn	Fe
		mg kg^−1^						g kg^−1^			mg kg^−1^						g kg^−1^		
1	1	0.25	0.40	3.3	21.1	1.5	11.3	0.12	0.08	10.0	ND	0.95	2.6	19.7	ND	13.8	0.08	0.10	5.9
2	1	1.8	6.6	6.8	33.6	14.7	43.9	0.13	0.14	30.0	0.55	0.55	0.76	12.5	0.61	25.9	0.11	0.15	1.6
3	1	1.9	28.7	17.7	24.3	8.5	46.2	0.18	0.77	25.3	0.55	4.1	3.7	23.3	0.76	67.8	0.16	0.32	3.9
3	2	1.1	41.5	5.2	19.2	2.9	23.0	0.13	1.1	12.6	0.40	4.4	2.7	10.4	1.1	14.2	0.15	0.26	2.8
4	1	0.60	2.8	4.0	14.9	5.8	16.0	0.11	0.23	11.4	ND	0.25	1.6	5.7	0.21	11.1	0.07	0.11	1.2
5	1	0.30	4.9	4.0	40.3	2.5	15.6	0.15	1.3	6.1	ND	0.60	1.1	7.1	ND	13.0	0.09	0.32	1.2
6	1	0.30	12.8	4.7	16.5	4.3	27.3	0.11	5.6	7.3	ND	1.4	1.5	7.8	ND	7.8	0.10	0.48	2.0
6	4	0.65	2.8	2.2	4.9	5.6	12.1	0.07	0.17	7.9	0.15	0.70	3.6	12.4	1.8	22.5	0.11	0.27	2.2
7	3	0.55	20.0	7.4	20.4	7.2	19.7	0.38	4.9	7.8	ND	0.35	0.11	8.0	0.01	8.1	0.08	0.25	0.30
8	7	0.20	10.4	4.2	16.5	0.96	20.9	0.04	2.2	7.2	ND	0.35	0.21	4.1	ND	4.0	0.04	0.33	0.35
9	3	0.45	7.7	5.7	15.0	3.0	12.6	0.05	2.2	7.8	ND	0.95	1.3	4.10	0.31	6.7	0.04	0.28	0.93
9	4	0.45	30.9	4.0	16.3	4.9	17.2	0.11	1.6	4.5	ND	0.25	0.41	6.5	ND	8.0	0.07	0.16	0.33
9	6	0.15	6.3	4.5	12.6	1.7	11.0	0.06	1.1	4.5	ND	0.55	1.60	8.0	ND	5.3	0.04	0.17	1.6
10	3	0.25	12.1	3.9	14.6	2.8	18.8	0.06	2.0	11.4	ND	1.5	0.76	4.9	ND	5.3	0.04	0.39	1.5
10	4	0.15	10.6	6.3	13.5	2.0	21.8	0.16	1.9	8.4	ND	0.45	0.46	7.1	ND	9.0	0.05	0.24	0.78
10	5	0.05	5.50	2.60	35.20	0.31	8.80	0.12	0.74	3.32	ND	0.55	0.51	8.4	ND	7.9	0.08	0.87	1.0
Mean		0.56	12.7	5.4	19.9	4.3	20.4	0.12	1.6	10.4	0.10	1.1	1.4	9.4	0.30	14.4	0.08	0.29	1.7
Median		0.37	9.0	4.3	16.5	2.9	18.0	0.12	1.2	7.8	-	0.58	1.2	7.9	-	8.6	0.08	0.26	1.3
**SD**		0.55	11.7	3.6	9.3	3.6	10.8	0.08	1.6	7.3	0.20	1.3	1.2	5.4	0.51	14.9	0.04	0.18	1.5

*ND* not detected

In the shoot of the plants, the concentrations of PTEs followed the same order when compared with the roots, with the inversion of Co and Cr (Table 2), in means followed by standard deviation in descending order (mg kg^−1^): Mn (292.3 ± 178.6) > Zn (82.5 ± 35.9) > Cu (14.4 ± 14.9) > Ni (9.4 ± 5.2) > Cr (1.4 ± 1.1) > Co (1.1 ± 1.2) > Pb (0.30 ± 0.50) > Cd (0.10 ± 0.19) and Fe (1,719.1 ± 1,417.6). The highest concentrations of PTEs in the shoot were observed in S1 for Fe (5,914.8 mg kg^−1^) at P1 and for Cd, Cr, Ni, Zn, and Cu (0.55, 3.7, 23.3, 158.7, and 67.8 mg kg^−1^, respectively) at P3. However, Co (4.4 mg kg^−1^) was found in S2 at P3, Pb (1.8 mg kg^−1^) in S4 at P6, and Mn (867.4 mg kg^−1^) in S5 at P10 (Table 2).

### Correlation analyses

There was a higher number of correlations between the parameters and the PTEs in the sediments, followed by the macrophytes and finally in the water (Table 3). In the correlations of the parameters in surface water samples, positive correlations were observed between EC-Mn (0.85); Fe–Mn (0.64) and Fe-Zn (0.69). And negative correlations between Mn and: Ca (−0.70); K (−0.68); Na (−0.74) and TDS (−0.68).

**Table 3:** Correlation matrix of parameters in water, sediments, macrophytes and potentially toxic elements in each system (*n* = 10)

	Parameters	Cd	Co	Cr	Cu	Mn	Ni	Pb	Zn
Water	pH	0.06	−0.40	−0.37	−0.10	−0.44	−0.49	−0.44	−0.15
	EC	−0.52	0.29	0.14	−0.20	0.85*	−0.11	0.15	0.15
	Turbidity	0.29	−0.39	−0.18	0.63	−0.01	0.02	−0.20	0.50
	Ca	−0.47	0.07	−0.30	−0.22	−0.70*	−0.04	−0.11	−0.54
	Mg	−0.53	0.129	−0.35	0.02	−0.37	0.07	−0.08	−0.33
	K	−0.53	−0.01	−0.35	−0.17	−0.68*	−0.19	−0.20	−0.41
	Na	−0.41	0.09	−0.26	−0.26	−0.74*	−0.06	−0.09	−0.60
	TDS	−0.52	0.10	−0.28	−0.18	−0.68*	−0.15	−0.22	−0.47
	DO	0.18	0.17	0.03	−0.22	−0.20	−0.13	−0.17	−0.35
	T	−0.52	0.16	0.05	0.45	0.20	0.33	0.26	0.48
	Fe	0.29	−0.16	0.22	0.46	0.64*	0.09	0.16	0.69*
	Parameters	Cd	Co	Cr	Cu	Mn	Ni	Pb	Zn
Sediment	Ca	0.89*	0.69*	0.65*	0.66*	0.48	0.70*	0.70*	0.82*
	Mg	0.84**	0.98**	0.50	0.73*	0.68*	0.89**	0.24	0.96*
	K	0.24	0.35	0.31	0.29	0.41	0.36	0.00	0.42
	SB	0.92*	0.81*	0.69*	0.69*	0.56	0.83*	0.54	0.89*
	CEC	0.92*	0.81*	0.69*	0.69*	0.56	0.83*	0.54	0.89*
	P	−0.42	−0.08	−0.52	−0.37	0.26	−0.18	−0.70*	−0.18
	pH	0.08	−0.18	−0.09	−0.19	0.33	−0.07	0.18	−0.08
	H + Al	−0.51	−0.39	−0.39	−0.46	0.16	−0.35	−0.16	−0.42
	OM	0.28	−0.01	0.26	0.01	0.29	0.12	0.04	0.02
	V%	0.99*	0.88*	0.73*	0.76*	0.53	0.90*	0.42	0.92*
	Clay	0.71*	0.37	0.90*	0.71*	0.01	0.49	0.89*	0.56
	Silt	0.73*	0.83*	0.45	0.77*	0.57	0.70*	0.45	0.90*
	Sand	−0.80*	−0.6	−0.88*	−0.90*	−0.27	−0.66*	−0.84*	−0.79*
	Fe	0.97*	0.81*	0.64*	0.67*	0.55	0.83*	0.51	0.86*
	Parameters	Cd	Co	Cr	Cu	Mn	Ni	Pb	Zn
Macrophyte	Ca	−0.28	−0.73*	−0.49	−0.26	−0.46	−0.06	−0.33	−0.52*
	Mg	−0.13	−0.61*	−0.33	−0.25	−0.23	−0.28	−0.08	−0.50
	P	0.42	−0.28	0.05	0.57*	−0.17	0.25	0.49	0.47
	K	0.31	0.76*	0.42	0.27	0.15	0.18	0.37	0.44
	S	0.56*	0.35	0.40	0.38	0.19	0.15	0.48	0.53*
	Fe	0.59*	0.73*	0.88*	0.55*	−0.07	0.69*	0.38	0.55*

* Correlation is significant at the 0.05 level (2-tailed)

In sediments, positive correlations were observed between some PTEs and Ca, Mg, SB, CEC, V%, Clay, Silt and Fe, and negative with Sand. Mn correlated only with Mg and Pb was the only PTE to correlate negatively with P. In macrophytes Fe correlated positively with some PTEs, as well as P-Cu (0.57), K-Co (0.76), S-Cd (0.56) and S-Zn (0.53). Negative correlations were observed Ca-Co (−0.73), Ca-Zn (−0.52) and Mg-Co (−0.61).

### Bioconcentration factor (BCF) and translocation factor (TF)

**Fig. 5:**
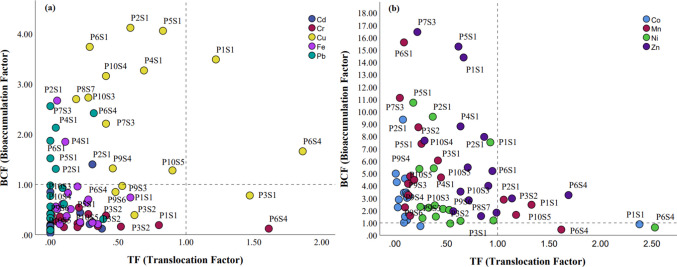
Scatter plot of Bioaccumulation Factor (BCF) x Translocation Factor (TF) for Potentially Toxic Elements (PTEs) in aquatic macrophytes collected from the Verruga River: Cd, Cr, Cu, Fe and Pb (**a**) and Co, Mn, Ni and Zn (**b**)

In descending order, the mean values for the Bioconcentration Factor (BCF) are expressed as follows: Mn > Fe > Zn > Ni > Cu > Co > Cr > Pb > Cd (Fig. 5). The highest values of BCF for Cd, Co and Fe was from P2S1 (*H. reniformis* at point 2); Pb was from P7S3 (*L. octovalvis*) and P6S4 (*P. stratiotes*), widespread in *H. reniformis*; Mn from *H. reniformis*; Zn from *L. octovalvis* and *H. reniformis*; Ni from *H. reniformis*; and Cu from P1S1 (*H. reniformis* at point 1).

The mean values of Translocation Factors (TF) for the PTEs followed the following descending order: Zn > Cu > Ni > Mn > Cr > Co > Fe > Cd > Pb (Fig. 5). The elements that had TF > 1 were Co, Cr, Cu, Mn, Ni, and Zn. The species *E. crassipes* (S4) stood out for having the highest TF for most elements: Cr (1.61); Mn (1.62); Ni (2.53); Zn (1.69); and Cu (1.86). Meanwhile, the species *L. peploides* (S2) for the elements Cd (0.38) and Pb (0.39), and the species *H. reniformis* (S1) for the elements Co and Cu at point 1 (P1).

## Discussion

About guidelines for drinking‐water quality naturally occurring, the limits for Pb (0.01 mg L^−1^) and Mn (0.08 mg L^−1^) have been exceeded (WHO 2022). Chemicals derived from the rocks and soil through which water percolates or over which it flows, some quality parameters should be considered when setting national standards (WHO 2022). The resolution of the National Environment Council—CONAMA RE 357/2005 (Brasil 2005) has classes (I—more restrictive and IV—less restrictive) to establish the water according to the treatment carried out and the purpose for human consumption.

All the samples were on the range for the limits of the following water quality parameters: pH; Cd; Co; Cr; Mn and Ni (Brasil 2005). However, they presented variations for the following parameters set by the legislation: DO not less than 6 mg L^−1^ (Class I) and higher than 2 mg L^−1^ of O_2_ (Class IV), were observed at P9 (3.4 mg L^−1^) and P10 (1.8 mg L^−1^); turbidity up to 40 NTU (Class I) and up to 100 NTU (Class III), at P4 (45 NTU); Pb limit is 0.01 mg L^−1^ (Class I) and 0.033 mg L^−1^ (Class III), at P2 (0.019 mg L^−1^) and P8 (0.012 mg L^−1^); dissolved Cu limit is 0.009 mg L^−1^ (Class I) and 0.013 mg L^−1^ (Class III), at P8 (0.017 mg L^−1^); dissolved Fe limit is 0.3 mg L^−1^ (Class I) and 5.0 mg L^−1^ (Class III), at P1 (0.47 mg L^−1^), P7 (0.34 mg L^−1^), P8 (1.3 mg L^−1^), P9 (0.73 mg L^−1^) and P10 (0.38 mg L^−1^) and total Zn limit is 0.18 mg L^−1^ (Class I) and 5.0 mg L^−1^ (Class III), at P8 (0.209 mg L^−1^).

Previous studies have reported concentrations above the limits set by current legislation for Cd and Pb in water (Santos et al. 2008). According to some parameters, the water of the Verruca River exceeds the quality limits for human consumption, after treatment (Class I), at point (P8) the concentration of Cu is superior to achieve the quality for irrigation, fishing and recreation (Class III) and at P10 dissolved oxygen is less than navigation and landscape harmony (Classe IV).

In sediments, the concentration of Cd, Cu and Ni exceeded the limits set by legislation from the resolution of the National Environment Council—CONAMA 454/2012 (Brasil 2012): Management of material to be dredged in freshwaters. It follows Canadian sediment quality guidelines for the protection of aquatic life (CCME 1995). They have reached the level 1 (Low probability of adverse effects on biota) for Cd (0.60 mg kg^−1^), Cu (35.3 mg kg^−1^) and Ni (18.0 mg kg^−1^) and the level 2 (High probability of adverse effects on biota) for Cd (3.5 mg kg^−1^) (Brasil 2012). According to Burton Junior (2002) Ni (> 18.0 mg kg^−1^), Cr (> 26.0 mg kg^−1^) and Cu (> 35.7 mg kg^−1^) reached the low effects levels and Cd (> 3.0 mg kg^−1^) exceeded the toxic effect threshold at P3.

Some aquatic macrophyte species exceeded the sufficient levels in shoots and the values were between the toxic ranges for Cu (20—100 mg kg^−1^), Zn (0.1—0.4 g kg^−1^), Ni (10—100 mg kg^−1^) and Mn (0.4—1.0 g kg^−1^) (Kabata-Pendias and Pendias 2001). *H. reniformis* at point 3 (P3S1) demonstrated great capacity of tolerance for Cu (67.8 mg kg^−1^), Zn (0.16 g kg^−1^) and Ni (23.3 mg kg^−1^). For Mn, *H. reniformis* at point 6 (P6S1) with 0.48 g kg^−1^ and *M. aquaticum* at point 10 (P10S5) with 0.87 g kg^−1^ were the highest concentrations.

The low concentrations of elements in the middle part of the basin indicate a self-purification process in the Verruga stream. This can be attributed to natural aeration in the area (Santos et al. 2008). Previous studies in the basin highlight that elements such as Cu, Zn, and Cd were observed when extracted by the water-soluble fraction, indicating their easy release from sediment to the water column (Santos et al. 2008). The weak correlations among PTEs in water show that metals are regulated by a mix of geochemical support and relationships, rather than by a single cause, and the strong correlations in sediments signifying that PTEs have the same sources of origin and similar behavior during transport (Bera et al. 2022).

Point P3 generally had the highest concentrations of Ca, Mg, and Na and the lowest pH value in the surface water. Additionally, it exhibited the highest concentrations of Cu, Zn, Fe, SB, CEC and V% in the sediments (likely due to the higher clay content). The sorption–desorption behavior of PTEs in aquatic environments is complex, and the processes are regulated by continuous interactions between surface water and sediments (Miranda et al. 2021). The dispersion of contaminants toward surface water can be reduced by clayey sediment (Förstner 2004).

Clays and oxide minerals promote the transfer of PTEs from surface water to sediments (Miranda et al. 2021). Cation exchange capacity (CEC) plays a role in the sorption and desorption of PTEs. A positive value means sorption to sediments. An increase in CEC favors the transfer of PTEs from water to sediments (sorption), attributed to clay concentrations, oxide minerals, and nutrients (Miranda et al. 2021). The presence of Fe oxides provides a reaction surface for sorption processes, allowing PTEs to bind and become immobilized. Increasing the solubility of Fe may have indirect effects on the mobility of PTEs, releasing them from the solid phase to water, Fe may re-precipitate as oxides and can bind the PTEs back into the solid state (Poting et al. 2021).

The electrical conductivity (EC) of the aqueous influences the concentrations of dissolved ions, which can be exchanged by PTEs in the solid phase. Higher EC in the surface water was associated with higher Mn concentrations, implying that an increase in ion concentrations is likely to increase the transfer of Mn from sediments to the aqueous phase (desorption) (Miranda et al. 2021). The concentration of Pb suggests it is influenced by total phosphorus. Higher phosphorus availability increases opportunities for the formation of relatively more stable complexes, such as metal phosphates. Pb adsorption to sediments due to its particular ability to form strong interactions with phosphates (Coz et al. 2007; Seshadri et al. 2017; Shen et al. 2020).

The behavior of PTEs depends on the nature and quantity of the elements. As for plants, absorption is related to species-specific mechanisms developed. Some PTEs are essential to the normal growth of plants and many chemicals including fungicides, pesticides, and herbicides contain Cu, Zn, Fe and Mn. Some trace metals such as Cd and Pb enter the soil as impurities of fertilizers (Antoniadis et al. 2017; He et al. 2005). Phosphorus (P) and Sulphur (S) can increase the uptake of some metals by plants by enhancing metal mobility and facilitates phyto-extraction. Application of P increases the hyperaccumulator potential of the plant and can be useful in jute plants grown in Cu-contaminated soil (Liu et al. 2023b; Saleem et al. 2020; Zhou et al. 2020;). The soil pH decreases with S, and solubility of PTEs increases, favoring the desorption from soil, and for increasing Cd and Zn accumulation in plants (Çimrin et al. 2007; Cui et al. 2004; Dede and Ozdemir 2016). The correlations between the PTE and nutrients in this study may be influenced by the chemical nature of PTEs.

The accumulation order of PTEs followed a similar order to that of Vardanyan and Inglore (2006) for the analyzed elements, with higher accumulations for Fe, Mn, Zn, and Cu, and lower for Pb and Cd. Although Cd and Cr were not detected in surface water, concentrations were observed in the roots, mainly *H. reniformis* (S1): Cd (1.9 mg kg^−1^) and Cr (17.7 mg kg^−1^), both at P3. Despite *H. reniformis* having high metal concentrations in the roots and, therefore, high bioconcentration factor (BCF) indices, translocation factors (TF) were mostly less than one, except for Co (TF = 2.4), Mn (TF = 1.3), and Cu (TF = 1.5), suggesting that this species has phytostabilizing properties (Haghnazar et al. 2021).

The species *E. crassipes* had the highest TF indices, characterizing it as a phytoextractor (Dhir et al. 2009). *E. crassipes* can be used as an ecological indicator that shows a direct response to metal concentrations in water (Singh et al. 2017). Floating macrophytes like *P. stratiotes* and *E. crassipes* are used for contaminant removal, proving to be effective in domestic wastewater treatment. Results demonstrate that *E. crassipes* can be considered a moderate accumulator of Cd and Zn (Ansari et al. 2020). Some species reported in this study are observed in the literature as phytoremediators and can reduce trace element concentrations in water: *E. crassipes* (Cr, Cu, Ni, Zn, Cd, and Pb); *P. stratiotes* (Cr, Fe, Mn, Cu, and Zn); *M. aquaticum* (Cu and Zn) (Dhir et al. 2009; Maine et al. 2004; Sanches Filho et al. 2015). Comparing metal content in macrophytes is challenging due to differences in sampling time, such as plant age and the presence of pollution sources. Additionally, metal data cannot be extrapolated from one species to another or even within the same species, largely due to different accumulation rates (Vardanyan and Inglore 2006).

Although *H. reniformis* may pose a problem in irrigated cultivation areas due to its high reproductive capacity and biomass production (Zaidan et al. 2020), and other native species compromise biota by presenting invasive characteristics, such as *E. crassipes*, *M. aquaticum*, and *P. stratiotes*, and exotics like the *Ludwigia genus*, they are marketed in Brazil at low prices (Peres et al. 2018). However, species like *E. crassipes*, *H. reniformis*, *Ludwigia* spp., *M. aquaticum* present a suitable option for improving contaminated effluents (Sipaúba-Tavares and Braga 2008).

In eutrophic systems, locally high concentrations of metals often occur as a result of the strong reducing environment associated with anthropogenic discharges (Vardanyan and Inglore 2006). As the concentration of PTEs such as Cr, Cd, Pb, and Ni was lower compared to essential elements such as Ca, P, K, Mg, S, Fe, Mn, Zn, and Cu, the elevated concentrations of metals in aquatic macrophytes may not directly reflect the pollution level of the areas. A similar trend of heavy metal accumulation underscores the universal significance of these macrophytes in purifying the aquatic environment (Vardanyan and Inglore 2006).

The use of plants as biomonitors of aquatic system pollution is a complex process since environmental characterization can influence physiological processes and thus vary the rates of bioaccumulation and translocation of elements. The sediment composition is an important factor in the sorption and desorption of PTEs, as mineral phases influence other properties, such as the physical and chemical characterization of the environment. Organic matter immobilizes total dissolved solids (TDS) (degree of water mineralization), while a decrease in pH tends to release elements from sediments into the water column. Although total dissolved solids can render Fe and Mn unavailable, phosphorus similarly retains Ni, Pb, and Cr, and electrical conductivity tends to release Mn.

## Conclusion

High concentrations of potentially toxic elements reached different levels of adverse effects on environmental quality and exceeded guidelines for the protection of aquatic life: Cu, Mn, Pb and Zn (surface water); Cd, Cu and Ni (sediments) and Cu, Mn, Ni and Zn (plants).

The species *Heteranthera reniformis* is a good option for use in environmental biomonitoring in the Verruga River, as it has a high occurrence frequency, high bioaccumulation and translocation factors. Despite being a small sample, our results suggest that the species *Eichhornia crassipes* is capable of translocating high concentrations of Cr, Cu, Ni, and Zn to the shoot, potentially serving as phytoaccumulators. The species *Ludwigia octovalvis* can accumulate Pb and Zn in its roots and *Pistia stratiotes* can accumulate Pb.

Human consumption of water from the Verruga River is not recommended due to high concentrations of certain PTEs that exceed safety limits set by the World Health Organization. This study recommends the investigation of the indicated species during seasonal variation, such as research on bioavailable element contents in sediments based on a sequential extraction fractional investigation to recognize alternatives to be explored for its best bioremediation environment.

## Data Availability

The article contains the data that was utilized to bolster the study’s conclusions. The corresponding author can provide more details upon request.
